# Members of WRKY Group III transcription factors are important in TYLCV defense signaling pathway in tomato (*Solanum lycopersicum*)

**DOI:** 10.1186/s12864-016-3123-2

**Published:** 2016-10-07

**Authors:** Ying Huang, Meng-Yao Li, Peng Wu, Zhi-Sheng Xu, Feng Que, Feng Wang, Ai-Sheng Xiong

**Affiliations:** State Key Laboratory of Crop Genetics and Germplasm Enhancement, College of Horticulture, Nanjing Agricultural University, 1 Weigang, Nanjing, 210095 China

**Keywords:** TYLCV, WRKY Group III TFs, VIGS, Regulation mechanism, Tomato

## Abstract

**Background:**

Transmitted by the whitefly *Bemisia tabaci*, tomato yellow leaf curly virus (TYLCV) has posed serious threats to plant growth and development. Plant innate immune systems against various threats involve WRKY Group III transcription factors (TFs). This group participates as a major component of biological processes in plants.

**Results:**

In this study, 6 WRKY Group III TFs (SolyWRKY41, SolyWRKY42, SolyWRKY53, SolyWRKY54, SolyWRKY80, and SolyWRKY81) were identified, and these TFs responded to TYLCV infection. Subcellular localization analysis indicated that SolyWRKY41 and SolyWRKY54 were nuclear proteins in vivo. Many elements, including W-box, were found in the promoter region of Group III TFs. Interaction network analysis revealed that Group III TFs could interact with other proteins, such as mitogen-activated protein kinase 5 (MAPK) and isochorismate synthase (ICS), to respond to biotic and abiotic stresses. Positive and negative expression patterns showed that WRKY Group III genes could also respond to TYLCV infection in tomato. The DNA content of TYLCV resistant lines after *SolyWRKY41* and *SolyWRKY54* were subjected to virus-induced gene silencing (VIGS) was lower than that of the control lines.

**Conclusions:**

In the present study, 6 WRKY Group III TFs in tomato were identified to respond to TYLCV infection. Quantitative real-time–polymerase chain reaction (RT-qPCR) and VIGS analyses demonstrated that Group III genes served as positive and negative regulators in tomato–TYLCV interaction. WRKY Group III TFs could interact with other proteins by binding to *cis* elements existing in the promoter regions of other genes to regulate pathogen-related gene expression.

**Electronic supplementary material:**

The online version of this article (doi:10.1186/s12864-016-3123-2) contains supplementary material, which is available to authorized users.

## Background

Microbial pathogens, including fungi, bacteria, and viruses, have disrupted plant growth, development, and yields. For instance, tomato yellow leaf curly virus (TYLCV) adversely affects the growth and development of tomato. This virus contains a circular single-stranded DNA (ssDNA) with 2.7–2.8 kb molecules. The TYLCV genome is composed of six open reading frames (ORFs), virion sense strands V1 and V2, and complementary sense strands C1–C4 [1]. First identified in Israel in 1930, TYLCV has invaded and damaged new areas, including the Mediterranean, Asian countries, and the US [2].

TYLCV is transmitted by the whitefly *Bemisia tabaci* in a persistent and circulative manner [3]. Intact virions pass through the food canal in the stylet of *B. tabaci* and reach the esophagus; they then move through the plant phloem as *B. tabaci* inserts its proboscis and feeds on infected tomato leaves [4, 5]. Upon entering plant host cells, the DNA of TYLCV replicates via a rolling circular mechanism [6]. Host enzymes can convert the incoming geminivirus ssDNA to double-stranded DNA (dsDNA) that serves as a transcription template. A complex composed of ssDNA and coat protein (CP) forms and enters the host nucleus. Host nucleus provides the factors for viral genome replication and transcription. The ssDNA:CP complex can then be transferred to an adjacent cell via two proposed routes: one route involves PreCP, which can bind to the ssDNA:CP complex and target the cytoplasm via the plasmodesmata and the other route includes CP, which participates in the nuclear export of the ssDNA:CP complex [7]. After TYLCV is released into the phloem of a young leaf by the feeding *B. tabaci*, this virus can systemically invade most plant organs above and below ground within 1–2 weeks [8]. TYLCV has threatened up to 20 different plant species. TYLCV infection-induced damages have also been exacerbated because of the uncontrollable spread of *B. tabaci*.

WRKY transcription factors (TFs) family, named from a highly conserved WRKY domain, is a large TF family distributed widely among plants [9]. The WRKY domain contains approximately 60 amino acids, comprising a highly conserved short peptide WRKYGQK at the N-terminus, and a C_2_H_2_ or C_2_HC zinc-binding motif at the C-terminus [10]. WRKY TFs can be divided into three groups (Groups I, II, and III) on the basis of the type of zinc-finger structures and the number of WRKY domains. *SPF1*, which encoded a WRKY factor, was cloned in *Ipomoea batatas* in 1994 [11]. Since then, numerous WRKY genes have been identified in different species, including terrestrial plants and green algae [12]. For instance, 72 WRKY members have been found in *Arabidopsis* [13], 95 members have been detected in *Daucus carota* [10], 50 members have been identified in *Camellia sinensis* [14], 100 members have been documented in *Oryza sativa* [15] and 145 members have been observed in *Brassica rapa* ssp. *pekinensis* [16].

Compared with Group I and II WRKY TFs, Group III TFs alter C_2_H_2_ to C_2_HC zinc-finger motif C-X7-C-X23-H-X_1_-C. Among the three groups, Group III has been considered the most adaptable and most advanced in monocot evolution [17, 18]. WRKY Group III TFs have also been identified in many plants: 13 members in *Arabidopsis* [19], 25 members in Chinese cabbage [16], 28 members in rice, 6 members in grape and 10 members in *Populus* [20]. WRKY Group III TFs participate in defense against pathogens. For example, resistance to *Magnaporthe oryzae* and *Xanthomonas oryzae* pv. *oryzae* in rice is strong because of *WRKY45* overexpression [21]. *ClWRKY70* and *FcWRKY70*, respectively identified in *Citrullus lanatus* and *Fortunella crassifolia*, are also implicated in plant disease resistance against pathogenic infections [22, 23]. Group III members in *Arabidopsis* are also involved in different plant defense signaling pathways; this phenomenon indicates that Group III TFs have evolved as a result of increasing biological requirements [19]. Furthermore, WRKY Group III TFs initiate transcriptional activation during pathogen invasion and function as essential components of plant innate immune systems, including basal defense and systemic acquired resistance [19, 24, 25]. WRKY TFs can bind to W/W-like box type *cis* elements or other *cis* elements in their promoters or in other gene promoters [26]. WRKY TFs also play a role in the interaction of salicylic acid (SA) and jasmonic acid (JA)-mediated signaling, which is involved in pathogen-associated molecular pattern (PAMP) triggered immunity and effector triggered immunity [27, 28]. WRKY TFs can also serve as major targets of perturbation-activated mitogen-activated protein kinase (MAPK) cascades [29, 30]. Therefore, certain WRKY Group III TFs significantly affect plant immunity through autoregulation, cross regulation, and transcriptional activation.

Tomato (*Solanum lycopersicum*) is an important vegetable that provides nutrients, including vitamins, dietary fiber, and sugars. As a model system, tomato has been used to investigate fruit development and ripening, domestication, and defense regulation [31–33]. Tomato was mostly produced in Asia from 1993 to 2014, followed by America and Europe (http://faostat.fao.org). Tomato production in Asia accounted for 51.2 % yield. However, pathogens have severely threatened tomato development and production. For instance, TYLCV infection has adversely affected various plant aspects, such as yield. In tomato, TYLCV infection has caused evident symptoms of mottling and yellowing of young leaves, upward curling of leaf margins, stunted growth, and flower abscission [34].

Tomato is a valuable vegetable worldwide but is prone to pathogenic infections, including TYLCV. Huang et al. conducted a genome-wide analysis of tomato WRKY genes, identified 81 WRKY TFs in tomato, and classified these TFs into three main groups, namely, Groups I, II, and III [35]. However, the roles of WRKY TFs, especially those in Group III, in tomato remain unclear. Our study aimed to obtain novel insights into the interaction of WRKY Group III TFs with TYLCV infection in tomato. Symptom characteristics, interaction network, expression profiles, and virus-induced gene silencing (VIGS) were also discussed to elucidate the response to TYLCV infection of WRKY Group III TFs. Our study further aimed to determine the functional mechanism of WRKY Group III TFs involved in defense against TYLCV and to improve the resistance to TYLCV infection of tomato.

## Methods

### Plant material and TYLCV infection

Derived from T5678161-1-1-2-2 and T07-018, ‘Zheza-301’ was bred to resist to TYLCV infection with the Ty-2 locus. ‘Jinpeng-1’, a hybrid of Holland tomato cultivar 99-13A and 9708B from America, was susceptible to TYLCV infection [6]. Seeds of ‘Jinpeng-1’ and ‘Zheza-301’ were obtained from Xi’an Jinpeng Seed Co., Ltd. and the Institute of Vegetables, Zhejiang Academy of Agricultural Sciences, respectively [6]. The seeds of TYLCV resistant tomato cultivar ‘Zheza-301’ and TYLCV susceptible cultivar ‘Jinpeng-1’ were sown in an artificial chamber (Nanjing Agricultural University (32°02’N, 118°50’E)) at 25 °C/18 °C for 12 h/12 h conditions with 300 μmol · m^−2^ s^−1^ light intensity.

Tomatoes were grown in hole trays (6 × 12 holes) filled with a mixture of vermiculite, organic solid, and perlite (1:2:1, v/v/v). Viruliferous whiteflies *Bemisia tabaci* (Hemiptera: Aleyrodidae) were allowed to feed on tomato plants in an insect-proof greenhouse in the Provincial Key Laboratory of Agrobiology, Jiangsu Academy of Agricultural Sciences (Nanjing, China). Upon reaching the two-leaf stage, tomato seedlings were exposed to viruliferous whiteflies, which fed on tomato plants to infect TYLCV. After 0, 2, 4, 6, 8, and 10 d of TYLCV infection, the leaves were collected and frozen in liquid nitrogen for RNA extraction. Tobacco (*Nicotiana tabacum*) from the genus *Nicotiana* was widely used as a premier plant cell biology model [36]. Tobacco seeds were also grown in the same artificial chamber to prepare for subcellular localization analysis.

### Sequence alignment and phylogenetic analysis of WRKY Group III TFs

The WRKY Group III TFs identified in rice, tomato, and *Arabidopsis* were downloaded from the Phytozome10. The WRKY Group III TFs sequences in Chinese cabbage were obtained from the *Brassica* Database (BRAD, http://brassicadb.org/brad/blastPage.php). PlantTFDB (Plant Transcription Factor Database) provided the WRKY TFs of other species [37]. Multiple sequence alignment was conducted using Clustal X, and MEGA 5 was used to conduct the phylogenetic tree based on the neighbor-joining method [38, 39].

### Exon/intron structure and promoter region analysis of WRKY Group III TFs

Gene Structure Display Server (GSDS v2.0, http://gsds.cbi.pku.edu.cn/index.php) was used to analyze the exon and intron structures of each WRKY Group III TF in rice, tomato, and *Arabidopsis* [40]. Approximately 1.5 kb of DNA sequence upstream from the codons of 6 WRKY Group III TFs in response to TYLCV infection was downloaded from the genome Solanaceae Genomics Network, (SGN, Release 2.3, http://solgenomics.net/organism/solanum_lycopersicum/genome). PlantCARE (http://bioinformatics.psb.ugent.be/webtools/plantcare/html/) was used to analyze the promoter region of WRKY Group III TFs [41].

### Identification of orthologous and paralogous gene and interaction network analysis of WRKY Group III TFs

OrthoMCL software was used to identify the orthologous and paralogous genes of WRKY TFs in tomato, *Arabidopsis*, and rice [42]. The relationship between orthologous and paralogous genes was displayed by Circos software [43]. The functional interaction network of WRKY Group III TFs in tomato was conducted. STRING software was responsible for the conduction of the interaction network [44].

### Subcellular localization analysis of WRKY Group III TFs in tomatoes

The full-length cDNA of *SolyWRKY41* and *SolyWRKY54* without stop codons were amplified into the pA7 vector by using specific primers in accordance with a previously described method to determine the subcellular localization of Group III TFs in tomato [10]. Empty vector pA7 containing 35S::GFP (green fluorescent protein) fusion protein was used as control. The constructed plasmids were then transferred into *N. benthamiana* leaves, which were placed on Murashige and Skooog (MS) medium via a biolistics procedure by using a helium-driven particle accelerator (PDS-1000, Bio-Rad). *N. benthamiana* leaves were then incubated for 24 h in the dark; the transient expression of GFP was then obtained using the LSM 780 confocal microscopy imaging system (Zeiss, Germany) [45]. The primers are shown in Table 1.

**Table 1:** Primers used in the text

Gene	Direction	Sequence(5'-3')	Function
*SolyWRKY41*	Forward	ATGGAGAAAGTTAAAAGTATGGA	Full lengths clone
	Reverse	TTAAATGAAGAATTCTTCAATGTC	
*SolyWRKY54*	Forward	ATGGATTGTGGATTCAATTATGAAT	Full lengths clone
	Reverse	TTATCTGAAAAAATCAGAGAAATTTG	
*SolyWRKY41*	Forward	CACCATCACCATCACGCCATGATGGAGAAAGTTAAAAGTATGGA	Subcellular localization
	Reverse	CACTAGTACGTCGACCATGGCAATGAAGAATTCTTCAATGTC	
*SolyWRKY54*	Forward	CACCATCACCATCACGCCATGATGGATTGTGGATTCAATTATGAAT	Subcellular localization
	Reverse	CACTAGTACGTCGACCATGGCTCTGAAAAAATCAGAGAAATTTG	
*SolyWRKY41*	Forward	GTGAGTAAGGTTACCGAATTCGCAACACCAAACCATAACGCTGAA	VIGS vector conduction
	Reverse	GGGACATGCCCGGGCCTCGAGAGGAATTTGAAATCGAAGTCGGAGT	
*SolyWRKY54*	Forward	GTGAGTAAGGTTACCGAATTCCCACAGAAACATAAACACAAGAAAC	VIGS vector conduction
	Reverse	GGGACATGCCCGGGCCTCGAGAGGAGAGACAAAAGATGGTGAATAA	
*SolyWRKY41*	Forward	GCAACACCAAACCATAACGCTGAA	RT-qPCR
	Reverse	AGGAATTTGAAATCGAAGTCGGAGT	
*SolyWRKY42*	Forward	AAGAAACTGTTCCTTTCACTCCACT	RT-qPCR
	Reverse	GAGCTGAACACAATACGATCCGATT	
*SolyWRKY53*	Forward	CCACAACCAACATCGCCAGAGAA	RT-qPCR
	Reverse	ACGGTGAATAGCCGCTACCTATCA	
*SolyWRKY54*	Forward	CCACAGAAACATAAACACAAGAAAC	RT-qPCR
	Reverse	AGGAGAGACAAAAGATGGTGAATAA	
*SolyWRKY80*	Forward	TCACTGTCCAACTTCAAACTCTACT	RT-qPCR
	Reverse	ACGGTCTTGCTTCTCCTTCTTCT	
*SolyWRKY81*	Forward	GCAATAGAAGGTTTAATTCGTGGTC	RT-qPCR
	Reverse	GTAGCGACGACGACATCAGA	
*SolyWRKY41*	Forward	AGTGACATATAAAGGAAGGCACAGT	RT-qPCR for VIGS
	Reverse	CCCTTCTCCACCAAATGAGGAATT	
*SolyWRKY54*	Forward	GCAAGTGCAGAGGTCTGATGATGA	RT-qPCR for VIGS
	Reverse	TGTTGATGAAGTGTTGGCTGAGAAC	
*TYLCV02*	Forward	CGCCCGTCTCGAAGGTTC	RT-qPCR for VIGS
	Reverse	GCCATATACAATAACAAGGC	
*TYLCV01*	Forward	ATGTCGAAGCGACCAGGCGATATAAT	Detection for TYLCV DNA
	Reverse	TTAATTTGATATTGAATCATAGAAAT	
*Tubulin*	Forward	TGACGAAGTCAGGACAGGAA	Reference gene
	Reverse	CTGCATCTTCTTTGCCACTG	

### Construction of VIGS vector and agroinfiltration

The fragments of *SolyWRKY41* (262 bp) and *SolyWRKY54* (243 bp) genes were also amplified with specific primers and constructed into pTRV2 vectors from Antoine Bouteilly (Centre National de la Recherche Scientifique). Recombinant plasmids of *SolyWRKY41* and *SolyWRKY54* were then transformed into *Agrobacterium tumefaciens* strain GV3101 by electroporation, respectively. *Agrobacterium* GV3101 cells were cultured in liquid YEB medium with 50 mg/L rifampin and 50 mg/L kanamycin overnight at 28 °C; cells were then collected and resuspended in infiltration media (10 mM MgCl_2_, 10 mM MES, and 200 mM acetosyringone) with an OD (optical density) value of 2.0. After being cultured at 28 °C for 4 h, the cotyledons of two tomato cultivar seedlings were infiltrated into the *Agrobacterium* [34]. Agroinfiltration of pTRV1 and pTRV2 served as negative control and pTRV2-*PDS* with pTRV1 were used as positive control.

For VIGS experiments, new emerging leaves from ‘Zheza-301’ were collected to extract RNA and determine the expression level of the target genes via quantitative real-time–polymerase chain reaction (RT-qPCR) after seven days of agroinfiltration. Seedlings of ‘Zheza-301’ were then transferred into the insect-proof greenhouse to feed the virusliferous whiteflies. After one week, the DNA was extracted from TYLCV-infected leaves to determine TYLCV DNA accumulation. Primers of *SolyWRKY41* and *SolyWRKY54* for RT-qPCR were designed to anneal outside the region targeted for silencing to ensure that only the endogenous gene would be detected [46].

### Quantitative real-time PCR analysis

To detect whether the DNA of TYLCV accumulated in resistant and susceptible tomato cultivars, we conducted a PCR analysis by using primer TYLCV01-F/R (Table 1). For PCR analysis, DNA was extracted from the two tomato cultivars after they were exposed to TYLCV infection for approximately 7 days. For VIGS experiments, total RNA and DNA were extracted from non-silenced and silenced plant leaves by using RNA kit (RNA simple total RNA kit) and DNA kits (DNA secure Plant kit) (Tiangen, Beijing, China) in accordance with the manufacturer’s protocols. To detect TYLCV accumulation, specific primers (TYLCV02-F/R) were used to conduct the RT-qPCR analysis (Table 1).

For RT-qPCR, SYBR Premix *Ex Taq* kit (TaKaRa, Dalian, China), iQTM5 software, and iQTM5 Real-time PCR System were used to complete the following procedure: 95 °C for 30 s initially, followed by 40 cycles at 95 °C for 5 s; 60 °C for 30 s and melting curve analysis (61 cycles) at 65 °C for 10 s. Three technical repeats were performed with each RNA sample of two tomato cultivars. *A-Tubulin* was used to regulate the expression level. 2^-ΔΔ*C*T^ method was used to measure the RNA level, which were expressed relative to the *Tubulin* gene [47].

## Results

### Detection of TYLCV DNA accumulation in resistant and susceptible tomato cultivars

**Fig. 1:**
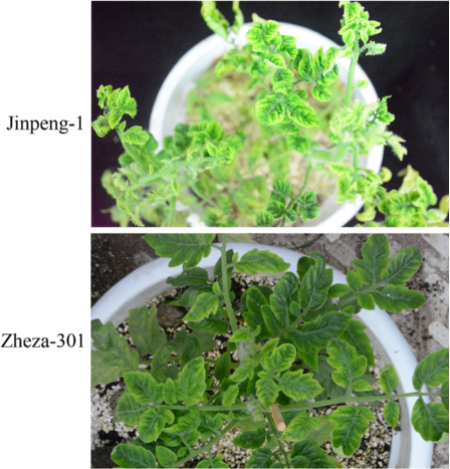
Symptom characteristics of ‘Zheza-301’ and ‘Jinpeng-1’ after TYLCV infection about 40 d

**Fig. 2:**
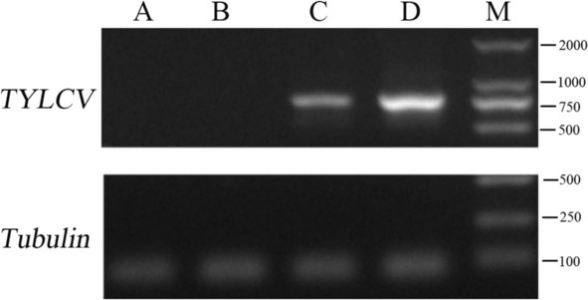
Detection of TYLCV DNA accumulation in control and TYLCV infection tomato cultivars by PCR. **a** Control plants: Zheza-301, **b** Control plants: Jinpeng-1, **c** Treatment plant: Zheza-301 after TYLCV infection at 7 d, **d** Treatment plant: Jinpeng-1 after TYLCV infection at 7 d, **m** marker

Two tomato cultivars (‘Zheza-301’ and ‘Jinpeng-1’) showed different symptoms after TYLCV infection. ‘Jinpeng-1’ was susceptible to TYLCV infection and the symptom in ‘Jinpeng-1’ was obvious than in ‘Zheza-301’ during the process of TYLCV infection. After TYLCV infection for 40 d, ‘Jinpeng-1’ showed more obvious symptom than ‘Zheza-301,’ which showed yellow and curly leaves, dwarfed plants, withered flowers, and small and shriveled newly formed leaves (Fig. 1). PCR analysis detected TYLCV DNA accumulation in both tomato cultivars. As shown in Fig. 2, in the control plants, there was no TYLCV accumulation in either ‘Zheza-301’ or ‘Jinpeng-1’. After TYLCV infection for 7 d, expression of TYLCV DNA was detected both in ‘Jinpeng-1’ and ‘Zheza-301’. There was higher expression of TYLCV DNA in ‘Jinpeng-1’than in ‘Zheza-301’.

### Members of tomato WRKY Group III TFs are involved in response to TYLCV infection

Comparative transcriptome profiling of resistant and susceptible tomato cultivars in response to TYLCV infection has been conducted by Chen et al. [48]. According to the transcriptome database (SRP028618), 6 WRKY TFs (SolyWRKY41, SolyWRKY42, SolyWRKY53, SolyWRKY54, SolyWRKY80, and SolyWRKY81) were identified to respond the TYLCV infection. The expression levels of these 6 WRKY genes in susceptible tomato cultivar were down regulated; by contrast, their expression did not significantly differ in the resistant tomato cultivar. These 6 WRKY TFs were classified into Group III according to the phylogenetic tree analysis, suggesting the importance of Group III TFs in the plant-pathogen interaction.

### Phylogenetic analysis of WRKY Group III TFs

**Fig. 3:**
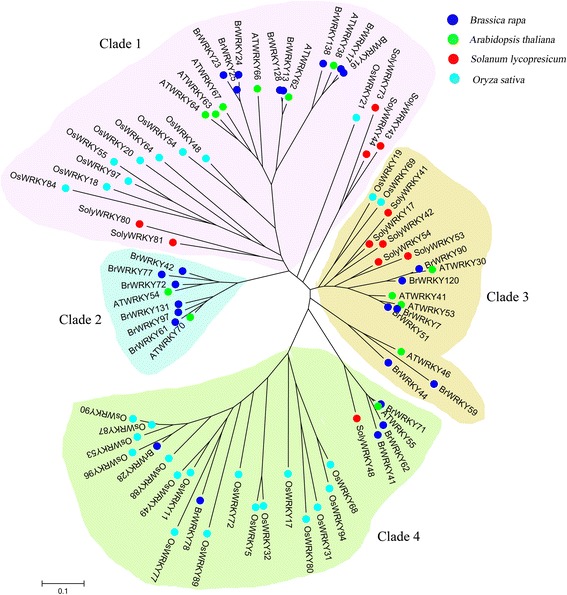
Phylogenetic tree of WRKY Group III TFs of tomato, *Arabidopsis*, Chinese cabbage, and rice

To investigate the phylogenetic relationship of WRKY Group III TFs in tomato, comparative analysis from three other sequenced plant genomes including the *Arabidopsis*, rice, and Chinese cabbage, was conducted using MEGA 5 (Additional file 1: Table S1) [16]. Clade 1 had the most Group III TFs members (28), followed by clade 4 (24), and clade 3 (17); clade 2 contained the least WRKY Group III TFs members (8) (Fig. 3). Clades 1, 3, and 4 contained WRKY Group III TFs in all 4 species. WRKY Group III TFs in tomato and rice were not classified into clade 2 which consisting of *Arabidopsis* and Chinese cabbage; this distribution may be related to some special circumstances (the split of different families in dicotyledon). Among those WRKY Group III TFs that responded to TYLCV infection in tomato, 2 members (SolyWRKY80, SolyWRKY81) and 4 members (SolyWRKY53, SolyWRKY54, SolyWRKY41, and SolyWRKY42) were classified into clades 1 and 3, respectively.

### Exon/intron structure analysis of each WRKY Group III TF

Gene structure including exon/intron structural diversification plays important roles in the evolution of multigene families [20]. To understand the structural diversity of WRKY Group III TFs, exon/intron structure analysis was performed on 81 WRKY TFs in tomato that were identified by Huang et al. [35]. As shown in Additional file 2: Figure S1, the exon/intron structures in the same group were similar. Group III TFs contained 11 members; 9 genes had two introns. For Group II, most genes had 3 exons in Group IIc and Group IId. By comparison, most Group IIa and IIb genes had at least 3. Of the 17 genes in Group IIe, 8 (*SolyWRKY18*, *SolyWRKY25*, *SolyWRKY39*, *SolyWRKY40*, *SolyWRKY36*, *SolyWRKY37*, *SolyWRKY38*, and *SolyWRKY40*) showed the same intron/exon structures.

**Fig. 4:**
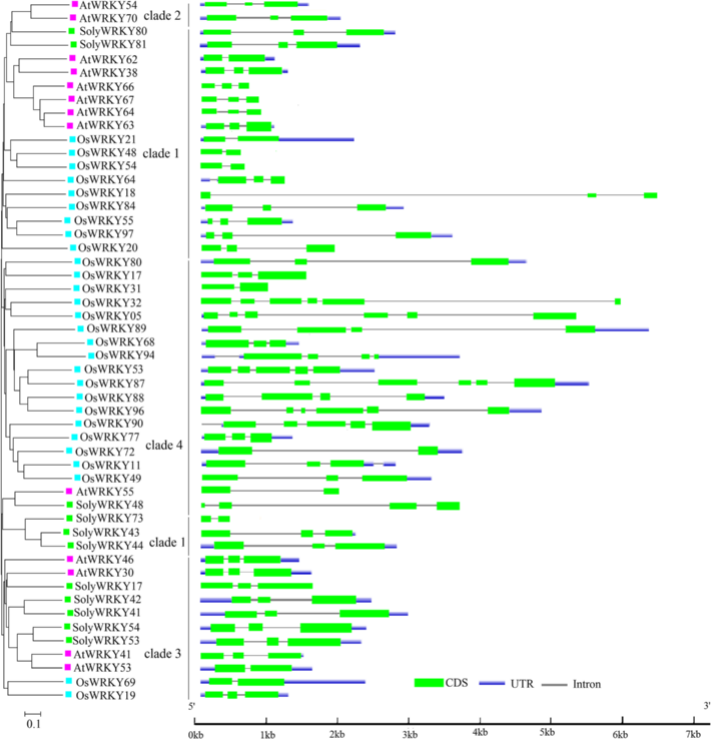
Exon/intron structure and phylogenetic relationship of WRKY Group III TFs from tomato, *Arabidopsis*, and rice. *Green rectangles* and *thin lines* represent the exons and introns, respectively. UTRs (untranslated regions) are showed by *thick blue lines*

Exon/intron organization maps from each Group III TF among *Arabidopsis*, rice, and tomato were generated (Fig. 4). Classified into 4 clades, 52 Group III TFs contained different exon numbers, ranging from 2 to 6. A total of 32 genes had 3 exons, followed by 2 exons (10), 4 exons (4) and 6 exons (4). *OsWRKY53* and *OsWRKY90* had 5 exons. Exon numbers of most genes in clade 4 were from 4 to 6. Among 11 WRKY Group III TFs in tomato, 9 genes contained 3 exons and 2 introns. *SolyWRKY73* of clade 1 only had 2 exons and 1 intron. *AtWRKY55*/*SolyWRKY48*, an orthologous pair according to the cluster at the terminal branch of the phylogenetic tree, showed the change of exon/intron numbers. Compared with *AtWRKY55*, *SolyWRKY48* gained 2 exons. The difference could also be found among those orthologous pairs *AtWRKY41*/*53*, *OsWRKY69*/*19*, and *OsWRKY53*/*87*. During WRKY Group III TFs evolution, exon/intro losses and gains have always occurred, which may be associated with the functional diversity among closely related Group III TFs.

### Promoter region analysis of SolyWRKY Group III TFs in response to TYLCV infection

**Fig. 5:**
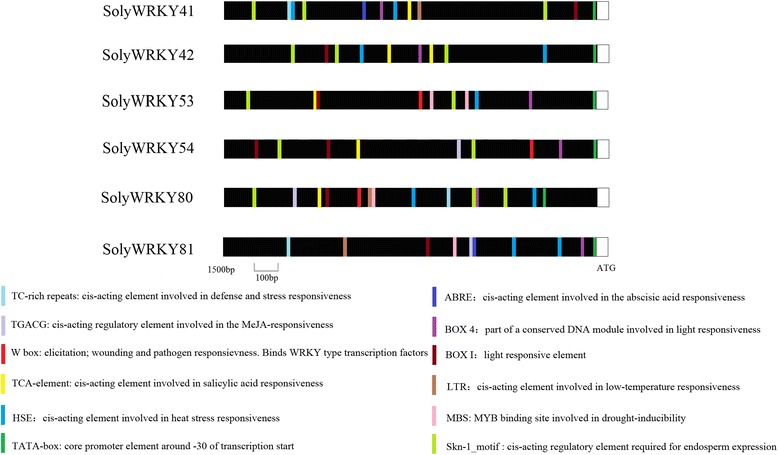
Protomer region analysis of six SolyWRKY Group III TFs involved in TYLCV defense. Different colors indicate different *cis* elements existing in the promoter region

In WRKY TF promoters, a series of W or W-like boxes suggested complex interaction of WRKY TFs with each other by binding with W-box elements [49, 50]. Some WRKY proteins could regulate their own gene expression by binding with *cis* elements in their own promoters [51, 52]. To determine if auto-regulation and cross-regulation existed in WRKY Group III TFs, promoter region, a sequence that could regulate and initiate gene transcription, was identified using approximately 1.5 kb DNA sequences upstream from the codons of 6 SolyWRKY Group III TFs (Fig. 5). Approximately 12 *cis* elements were found in most SolyWRKY Group III TFs, including W-box, TATA-box, TC-rich, and HSE-element. Some basic elements, including Box 4 (part of a conserved DNA module involved in light response), Box I (light-responsive element), Skin 1 motif (*cis*-acting regulatory element required for endosperm expression), and TATA elements (core promoter element around −30 of transcription start), existed in the promoter regions of all 6 SolyWRKY Group III TFs.

In the study, elements related to hormone regulation, such as ABRE (*cis*-acting element involved in abscisic acid response), TCA (*cis*-acting element involved in salicylic acid response), and TGACG element (*cis*-acting element involved in the MeJA response) were identified in Group III TF promoter region. *Cis* elements related to abiotic stress were found in several SolyWRKY TFs promoter regions; for example, HSE/LTR-elements, which are involved in high/low temperature responsiveness, suggested that upstream genes could regulate WRKY gene expression by binding to the corresponding *cis* elements. Several SolyWRKY TFs (SolyWRKY53, SolyWRKY54, and SolyWRKY80) contained the W or W-box element in their promoter regions, suggesting that these SolyWRKY TFs could be regulated by interaction with each other or by auto-regulation (Fig. 5).

### Microsynteny analysis of WRKY Group III TFs

**Fig. 6:**
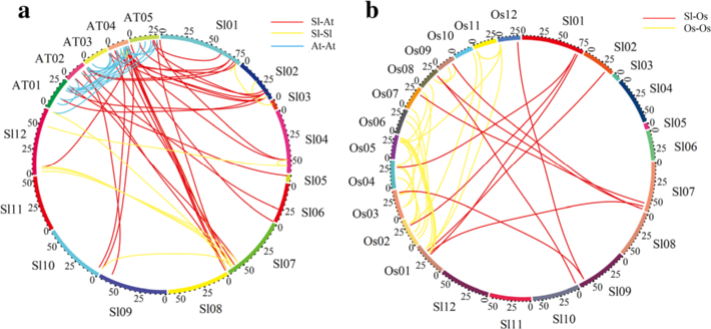
Comparative analysis of WRKY TFs in tomato, *Arabidopsis*, and rice. **a** Microsynteny of WRKY TFs across tomato and *Arabidopsis*. Tomato chromosomes label Sl01- Sl12. AT01-AT05 represents the *Arabidopsis* chromosomes. *Red lines* represent the orthologous WRKY genes among tomato and *Arabidopsis*. *Yellow* and *blue lines* represent paralogous WRKY genes in tomato and *Arabidopsis*, respectively. **b** Microsynteny of WRKY TFs across tomato and rice. Tomato chromosomes label Sl01- Sl12. OS01-Os12 represents the rice chromosomes. Red lines represent the orthologous WRKY genes among tomato and *Arabidopsis*. *Yellow lines* represent paralogous WRKY genes rice

Two consecutive genome triplications have happened in the *Solanum* lineage [53]. In the study, *Arabidopsis* and rice were selected to make the microsynteny with tomato using whole-genome sequence according to the homologous gene locations. First, we analyzed the relationship of WRKY TFs in the three species. As shown in Fig. 6, 47 and 12 pairs of orthologous WRKY TFs were identified between tomato and *Arabidopsis* and between tomato and rice, respectively (Additional file 1: Table S2). Moreover, tomato, *Arabidopsis*, and rice contained 28, 19, and 39 paralogous WRKY TFs pairs (Additional file 1: Table S3).

**Fig. 7:**
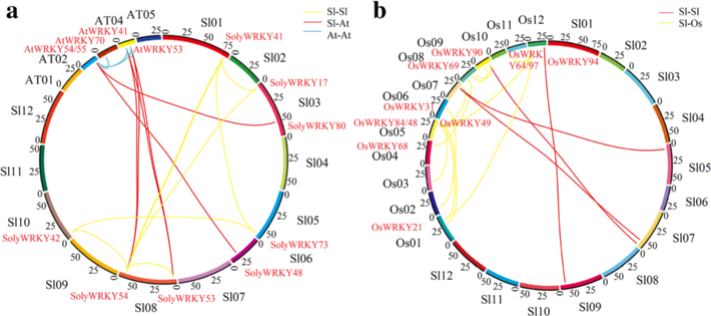
Microsynteny of WRKY Group III TFs in tomato, *Arabidopsis*, and rice. **a** Microsynteny of WRKY TFs across tomato and *Arabidopsis*. Tomato chromosomes label Sl01- Sl12. AT01-AT05 represents the *Arabidopsis* chromosomes. *Red lines* represent the orthologous WRKY genes among tomato and *Arabidopsis*. *Yellow* and *blue lines* represent paralogous WRKY genes in tomato and *Arabidopsis*, respectively. **b** Microsynteny of WRKY Group III TFs across tomato and rice. Tomato chromosomes label Sl01- Sl12. OS01-Os12 represents the rice chromosomes. Red lines represent the orthologous WRKY genes among tomato and *Arabidopsis*. *Yellow lines* represent paralogous WRKY genes rice

Eleven WRKY Group III TFs from tomato were distributed across 7 chromosomes. SolyWRKY43, SolyWRKY44, and SolyWRKY73 were located in chromosome 05. Chromosomes 03 and 08 each contained 2 members of WRKY Group III TFs, followed by chromosomes 01 (1), 06 (1), 09 (1), and 10 (1) (Additional file 3: Figure S2). To analyze the orthologous and paralogous relationships of WRKY Group III TFs between eudicots and monocots, the microsynteny analyses of *Arabidopsis*, tomato, and rice were conducted on the basis of WRKY Group III TF locations of orthology and paralogy gene pairs. As shown in Fig. 7, 7 collinear gene pairs were identified in the tomato genome. Four WRKY Group III TFs, including SolyWRKY80, SolyWRKY81, SolyWRKY48, and SolyWRKY43, from tomato were absent in any microsynteny. This finding indicated that independent duplication events, except whole genome duplication, occurred.

The corresponding interspecies relationships of microsynteny were also analyzed. Six orthologous gene pairs, namely, SolyWRKY53/AtWRKY53, SolyWRKY53/AtWRKY41, SolyWRKY54/AtWRKY53, SolyWRKY54/AtWRKY41, SolyWRKY48/AtWRKY55, and SolyWRKY80/AtWRKY54, were found between tomato and *Arabidopsis*. Four orthologous gene pairs existed between tomato and rice. This finding suggested that the relationship between tomato and *Arabidopsis* was closer than that between tomato and rice. Some collinear gene pairs were present in tomato and *Arabidopsis* but were absent in tomato and rice because rice diverged from the common ancestor of tomato and *Arabidopsis*. Among 11 SolyWRKY Group III TFs, SolyWRKY81 showed no linkage with other WRKY TFs, which may take part in the expansion of WRKY Group III TFs.

### Evolution of WRKY Group III TFs among different species

**Fig. 8:**
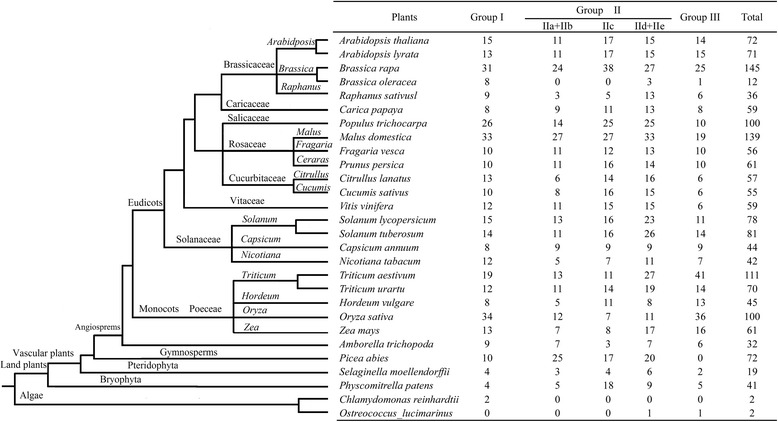
Comparison of WRKY family transcription factors among different plants

WRKY TFs are common not only in land plants but in algae as well [10]. To analyze the evolution of WRKY Group III TFs, a schematic of the phylogenetic tree was conducted (Fig. 8). A total of 1,722 WRKY TFs among 28 species were identified. As shown in Fig. 8, numbers of WRKY TFs in land plants are larger than in algae, which contain only 2 WRKY TFs in *Chlamydomonas reinhardtii* and *Ostreococcus lucimarinus*. In algae, *C. reinhardtii* showed no Group III TFs. WRKY Group III TFs exist in most land plants, except *Picea abies*. The numbers of WRKY Group III TFs in monocots were more than in eudicots. Percentage of Group III TFs in monocots was almost at 20.00 %–36.94 %. Group III TFs in *Triticum aestivum* occupied 36.94 % in monocots; by contrast, *A. lyrata* yielded the highest percentage (21.12 %) of Group III TFs in eudicots. These results indicated that numerous diversifications for WRKY TFs, particularly WRKY Group III TFs, occurred after the divergence of the monocots and eudicots. Notably, Group I TFs exists in all the land plants, suggesting that Group I may have evolved earlier.

### Subcellular localization analysis of SolyWRKY Group III TFs involved in TYLCV infection

**Fig. 9:**
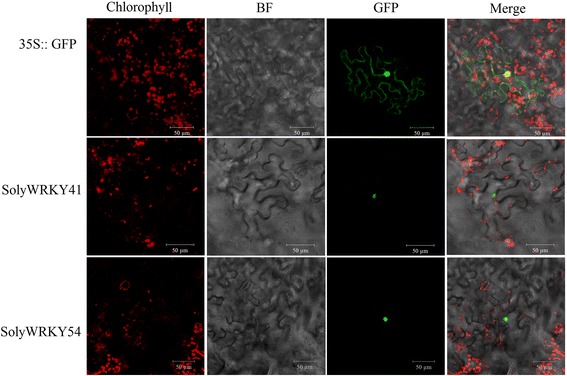
Subcellular localization of SolyWRKY Group III genes in response to TYLCV infection. Confocal images showing the localization of SolyWRKY Group III genes and terminal proteins transiently expressed in *N. benthamiana* leaves. *Bar* 50 μm. Chlorophyll: Image after chlorophyll autofluorescence; BF: Image after bright field. GFP: Image after green fluorescence. Merge represents the Image after merger of chlorophyll, bright field, and green fluorescence

In tomato, 4 out of 6 SolyWRKY Group III TFs in response to TYLCV infection were classified into clade 3. Two clade 3 TFs pairs (SolyWRKY53/54 and SolyWRKY41/42) were clustered together (Fig. 3). To determine the subcellular localization of SolyWRKY Group III TFs, constructions of two clade 3 members (SolyWRKY41-GFP and SolyWRKY54-GFP) and empty vectors containing 35S::GFP were introduced into the *N. benthamiana* leaves. *N. benthamiana* leaf cells of the control with 35S::GFP showed GFP fluorescence throughout the cell. In contrast, confocal laser scanning microscopy showed that SolyWRKY41-GFP and SolyWRKY54-GFP exhibited clear signals in the nucleus of the *N. benthamiana* leaf cells (Fig. 9). Results indicated that SolyWRKY41 and SolyWRKY54 were nuclear proteins in vivo.

### Interaction network of WRKY Group III proteins in tomatoes

**Fig. 10:**
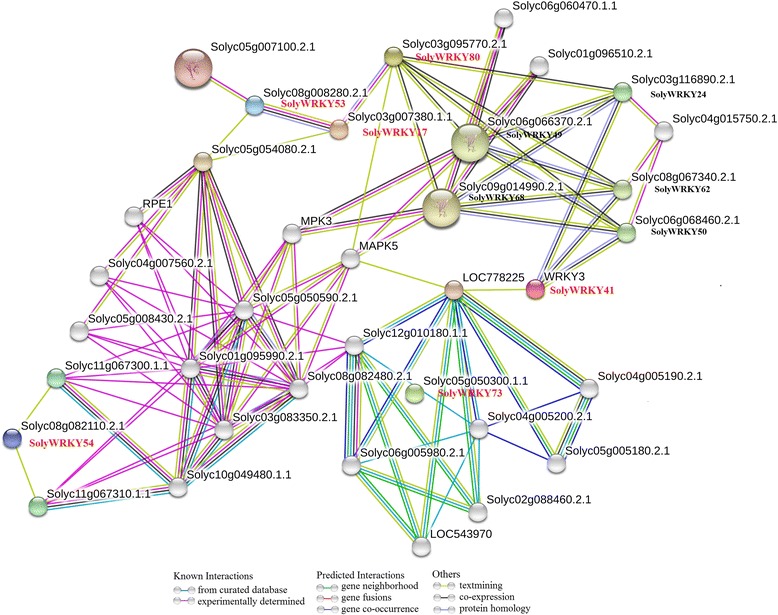
Interaction network of six Soly WRKY Group III TFs in response to TYLCV infection

Interaction network of WRKY Group III TFs was analyzed to further understand about the regulation mechanism. As shown in Fig. 10, 5 Group III proteins (SolyWRKY17, SolyWRKY41, SolyWRKY53, SolyWRKY54, and SolyWRKY80) showed interactions with other proteins in tomato genome. SolyWRKY80 showed co-expression relationships with 2 WRKY Group I proteins (SolyWRKY68 and SolyWRKY49), and 2 WRKY Group IIa proteins (SolyWRKY50 and SolyWRKY62). SolyWRKY80 could also interact with MAPK5. SolyWRKY41was identified to interact with other WRKY proteins (SolyWRKY24, SolyWRKY62, and SolyWRKY50), suggesting SolyWRKY80 and SolyWRKY42 had the similar functions in the regulation network. Isochorismate synthase (ICS, LOC778225), could interact with SolyWRKY41 protein. The WRKY Group III proteins, SolyWRKY80 and SolyWRKY53 showed significant correlations with SolyWRKY17.

### Expression profiles of SolyWRKY Group III genes in response to TYLCV infection

**Fig. 11:**
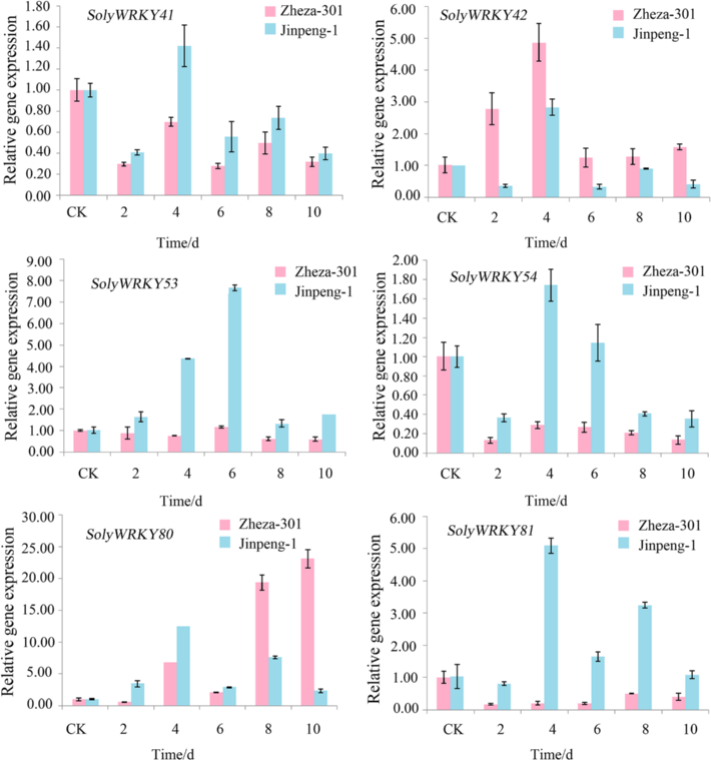
Expression levels of six Soly WRKY Group III genes after TYLCV infection in tomatoes

To elucidate the expression patterns of SolyWRKY Group III genes involved in TYLCV response in resistant and susceptible tomato cultivars, *SolyWRKY41*, *SolyWRKY42*, *SolyWRKY54*, *SolyWRKY53*, *SolyWRKY80*, and *SolyWRKY81* were selected for RT-qPCR analysis after TYLCV infection at different times in resistant and susceptible tomato cultivars (Fig. 11). The expression levels of 6 SolyWRKY Group III genes increased in the susceptible tomato cultivar ‘Jinpeng-1’ after TYLCV infection; and in the resistant tomato cultivar ‘Zheza-301’, 4 Group III genes showed down-regulation, except for *SolyWRKY42* and *SolyWRKY80*. In ‘Jinpeng-1,’ after 6 d of TYLCV infection, the expression level of *SolyWRKY53* increased by approximately 8.0-fold. The highest expression peaks of other five genes were observed after TYLCV infection for 4 d, with approximately 13-fold in *SolyWRKY80*, 5-fold in *SolyWRKY81*, and 3-fold in *SolyWRKY42* (Fig. 11). In ‘Zheza-301,’ *SolyWRKY80* showed rapid increase after 6 d and the expression peaked at 23-fold after TYLCV infection for 10 d. Expression of *SolyWRKY42* showed the highest expression level at 4 d with 5-fold.

### VIGS validation of WRKY Group III genes in response to TYLCV infection in tomatoes

To further analyze the function of SolyWRKY Group III genes in response to TYLCV infection, VIGS validation was conducted in resistant tomato cultivar ‘Zheza-301’. Two SolyWRKY Group III genes (*SolyWRKY41* and *SolyWRKY54*), classified in clade 3, were knocked down by the tobacco rattle virus (TRV) mediated VIGS system (Fig. 3) [54].

**Fig. 12:**
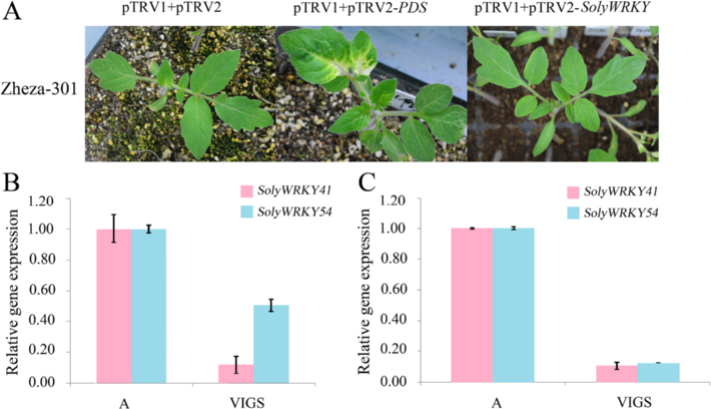
Validation of putative Soly WRKY Group III genes by virus induced gene silencing (VIGS). **a** Cotyledon agroinfiltration of pTRV vectors was conducted in the resistant cultivar ‘Zheza-301’ at the cotyledon stage. After three weeks, leaves with pTRV1 and pTRV2-SolyWRKY showed normal phenotype. Leaves of ‘Zheza-301’ with pTRV1 and pTRV2-PDS showed bleached areas. **b** The expression level of two SolyWRKY Group III genes in the VIGS treated plants one week after agroinfiltration. *A-Tubulin* (Solyc04g077020.2) was used as an internal reference. ‘A’ represented the ‘Zheza-301’ treated with pTRV1 and pTRV2 (negative control), ‘VIGS’ represented the ‘Zheza-301’ with pTRV1 and pTRV2-SolyWRKY. **c** The accumulation of TYLCV DNA in the SolyWRKY Group III genes silenced ‘Zheza-301’ plants. ‘A’ represented the ‘Zheza-301’ treated with pTRV1 and pTRV2 (negative control), ‘VIGS’ represented the ‘Zheza-301’ with pTRV1 and pTRV2-SolyWRKY

As shown in Fig. 12a, after agroinfiltration for 3 weeks, positive control plant with pTRV1 and pTRV2-PDS showed leached areas in leaves. Expression levels of *SolyWRKY41* and *SolyWRKY54* after silencing were decreased by approximately 20 % and 50 %, respectively, compared with those in the negative control (Fig. 12b). Meanwhile, TYLCV DNA accumulation was detected after TYLCV infection for one week. As shown in Fig. 12c, TYLCV DNA accumulation decreased in silencing *SolyWRKY41* and *SolyWRKY54*, compared with those in the control plants. Results indicated that *SolyWRKY41* and *SolyWRKY54* negatively regulated response to TYLCV infection.

## Discussion

Plants are constantly exposed to various adverse stress conditions, such as pathogen infection and abiotic stress, because of their sessile growth habit. During pathogen infection, genes could activate inducible defense responses in host plants by transcriptional regulation [55]. A large number of WRKY TFs that contain WRKY domains participate in pathogen infection and other treatments, such as SA, wounding, or senescence [56–58]. As an important group of WRKY TFs, Group III TFs are involved in plant innate immune systems; however, limited information is available regarding the biological functions of WRKY Group III TFs in response to TYLCV infection of tomato. In our study, expression analysis and VIGS-based knockdown demonstrated that WRKY Group III TFs functioned as positive and negative regulators in defense response against TYLCV infections in tomatoes.

### TYLCV infection in tomato cultivars

In this study, viruliferous whiteflies spread the virus by feeding on tomato leaves. Studies showed that whiteflies could carry and transmit TYLCV and other viruses, including cucurbit leaf crumple virus and tobacco curly shoot virus [59, 60]. PCR analysis was used to detect TYLCV DNA accumulation in tomato leaves. As shown in Fig. 2, compared with control plants, TYLCV DNA showed high expression levels in both two tomato leaves after TYLCV infection for 7 d. Numerous studies have conducted the analyses between plants and TYLCV infection using the whiteflies as the medium, suggesting their feasibility in conducting the analysis of plant-TYLCV interaction [3, 61–63].

### Gene structure and evolution of WRKY Group III TFs

Multigene family evolution can be driven by gene structural diversity [20]. Exon/intron organization mapping of WRKY Group III TFs from tomato, *Arabidopsis*, and rice was conducted to better understand the structural diversity. A total of 52 WRKY Group III TFs contained different numbers of exon/intron. Gene structural diversity existed in these three species. *AtWRKY53* and *OsWRKY69* in clade 3 contained 2 exons; by comparison, 3 exons existed in other WRKY Group III TFs in this clade. Similar exon/intron structures could be found in most recent paralogs. *AtWRKY55*/*SolyWRKY48*, an ortholog pair, showed change of exon/intron numbers, indicating the exon/intron loss or gain during the long evolutionary period [64]. Huang et al. have analyzed the conserved motifs of WRKY Group III TFs, which were also important to diversified functions of Group III proteins [35]. Different exon/intron structures existed among 11 SolyWRKY Group III TFs, such as SolyWRKY73 and SolyWRKY48, although similar motif structures were identified [35]. Differences in exon/intron structures and motif characteristics indicated functional diversity of WRKY Group III TFs.

To explore the evolution of WRKY Group III TFs, we first analyzed the relationship between tomato with *Arabidopsis* and rice by microsynteny. Difference in the orthologous gene pairs between tomato/*Arabidopsis* (6) and tomato/rice (4) indicated the closer relationship of tomato and *Arabidopsis*. Independent duplication also existed in the process of WRKY Group III TFs. During species evolution, gene duplication and loss have always taken place. A model diagram among plantae plants was then built to further analyze the origin and evolution of WRKY Group III TFs. As shown in Fig. 8, WRKY Group I TFs exist in almost all species, even in *C. reinhardtii*. However, Groups II and III TFs are identified in most land plants. These results showed that Group I had early origin, but Groups II and III TFs expanded with the evolution of higher plants [17, 65]. Compared with algae, land plants have a relatively large number of Group III TFs. Significant difference in the number of Group III TFs between monocotyledonous and dicotyledonous plants suggested higher function and more active duplication in monocots than in eudicots.

### WRKY Group III TFs involved in TYLCV infection in tomatoes

Six SolyWRKY TFs belonging to Group III are involved in response to TYLCV infection according to a previously described database [48]. The *cis*-regulatory elements were identified in the promoter regions of 6 SolyWRKY Group III proteins involved in different functions, such as abiotic stress (LTR, HSE elements), hormone regulation (ABER,TCA, and MeJA elements), and disease resistance (W-box elements). WRKY TFs could be regulated by other WRKY TFs and proteins with special binding to different *cis* elements. WRKY proteins could bind with W or W-box (C/T)TGAC(T/C) elements to regulate the pathogen defense-related genes [10]. In rice, 2 *cis* elements, PRE2 and PRE4 (harboring the W box), were identified from the promoter region of OsWRKY13, which could positively regulate *OsWRKY13* gene expression after pathogen-induced protein binding [66]. WRKY13 could bind to W-box in WRKY42 and regulate its expression [26]. There were W boxes existed in the promoter regions of SolyWRKY53, SolyWRKY54, and SolyWRKY80, suggesting these WRKY Group III TFs can participate in TYLCV defense process by autoregulation or cross-regulation with each other [49, 51].

In plant immunity, WRKY TFs could form a complex interconnected regulatory network at several different levels [49, 67]. In the study, an interaction network of SolyWRKY Group III proteins was conducted, which showed that Group III proteins could interact with a series of defense proteins. For example, MAPK5 could phosphorylate some TFs including WRKY to activate the transcription of other genes and interact with SolyWRKY80 [68, 69]. ICS is required to synthesize SA for plant defense [70]. SolyWRKY41 showed significant interaction with ICS by binding the W-box elements existed in the promoter region of ICS. Interaction relationships also existed among WRKY proteins, even in the same group. Two WRKY Group III proteins (SolyWRKY80 and SolyWRKY53) both showed co-expression with SolyWRKY17 (Fig. 10).

### Expression profiles of SolyWRKY Group III genes in response to TYLCV infection

In the study, 6 SolyWRKY Group III genes (*SolyWRKY41*, *SolyWRKY42*, *SolyWRKY53*, *SolyWRKY54*, *SolyWRKY80*, and *SolyWRKY81*) were selected to conduct RT-qPCR and VIGS validation analyses (Figs. 11 and 12). WRKY Group III genes in tomato could be implicated in TYLCV defense by inducing positive and negative expression patterns in resistant and susceptible tomato cultivars. Two clade 3 WRKY TFs (SolyWRKY41 and SolyWRY54) were chosen for subcelluar localization. Subcellular localization analysis by using tomato is not yet mature. Numerous studies on tomatoes have successfully used tobacco to conduct subcellular localization [71, 72]. SolyWRKY41 and SolyWRKY54 were located in the nucleus of the *N. benthamiana* leaf cells. Expression levels of *SolyWRKY41* and *SolyWRKY54* were decreased compared with those of the control after VIGS. The TYLCV DNA expression also decreased in the silencing *SolyWRKY41* and *SolyWRKY54* plants, suggesting *SolyWRKY41*and *SolyWRKY54* were down-regulated upon TYLCV infection in resistant tomato cultivar ‘Zheza-301.’

During the process of induced disease resistance, transcriptional regulation of defense gene expression appeared to play a central role [73]. Overexpression of WRKY TFs genes has been shown to confer disease resistance and induce a number of defense-related genes in systemic acquired resistance, including pathogenesis-related (PR) genes (*PR1*, *PR2*, and *PR5*) [74]. Overexpression of *AtWRKY70* increased the resistance to virulent pathogens and induced expression of SA-induced pathogenesis-related genes [73]. AtWRKY53 was implicated as positive regulators of senescence [75]. Overexpression of *WRKY12* resulted in reduced soft symptoms by *Pectobacterium carotovorum* in *Arabidopsis* and Chinese cabbage [76]. In tomato, silencing of *SlDRW1*, the defense-related WRKY1 gene, resulted in increased severity caused by *Botrytis cinerea*, suggesting the positive regulation of *SlDRW1*. Overexpression of WRKY TFs genes could induce a series of related protein expression to regulate resistance to abiotic and biotic stresses, as well as plant growth. In the study, *SolyWRKY42* and *SolyWRKY80* served as positive regulators in the defense from TYLCV infection process (Fig. 11). These genes increased the resistance in tomato by inducing expression of resistant-related genes or involving SA or JA signaling.

In *A. thaliana*, 13 Group III members out of 74 WRKY TFs could respond to different plant defense signaling pathways. Orthologous genes of SolyWRKY80, SolyWRKY81, SolyWRKY53, and SolyWRKY54, AtWRKY53 (At4g23810) and AtWRKY70 (At3g56400) were important in plant-pathogen interaction [19]. In addition to WRKY TFs, AP2/ERF TFs could respond to TYLCV infection, and 5 different SolyERF genes (*Soly19*, *Soly36*, *Soly106*, *Soly67*, and *Soly66*) are involved in TYLCV defense with a complex network through positive and negative expression [77]. Negative and positive regulations were both important for gene expression [78]. Wang showed that *SlybHLH131* was up-regulated in resistant tomato line and down-regulated in susceptible tomato line after TYLCV infection, which was similar to the expression patterns of *SolyWRKY53* and *SolyWRKY80* [79]. In TYLCV defense, SolyWRKY Group III TFs could serve as positive and negative regulators in the plant defense network and help orchestrate a broad and spatially controlled response.

### A probable function mechanism of WRKY Group III involved in TYLCV defense

**Fig. 13:**
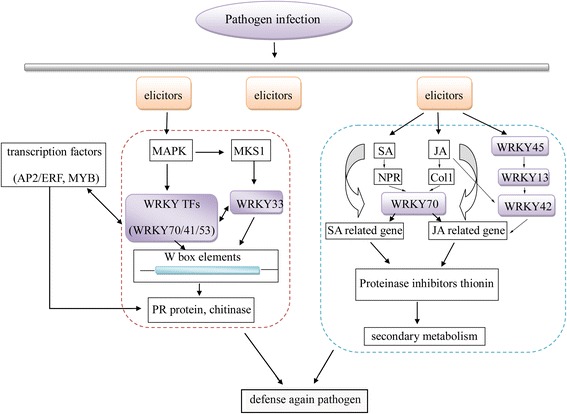
A possible function network of SolyWRKY Group III TFs in response to TYLCV infection

Transcriptional regulation of defense gene expression is pivotal in inducing disease resistance [80]. A probable regulation network on WRKY-TYLCV infection was conducted [26, 67, 68, 73, 81]. During pathogen infection, WRKY Group III TFs may respond to adverse stress by participating in various signaling pathways, such as hormone signaling, pathogen-induced defense program (Fig. 13). There is complex connection between MAPKs and TFs including WRKY [10, 76]. A MAPK cascade may activate the expression of some TFs (AP2/ERF, WRKY) [68]. SolyWRKY80 showed significant interaction with MAPK5, which was identified to involve in the defense against fungal and viral attacks [82, 83]. AP2/ERF TFs participate in many processes in plant development and induce PR protein expression [81]. Autoregulation and cross-regulation also occur in WRKY TFs by binding to specific *cis* elements, such as W-box (Fig. 5). WRKY45-2, WRKY13, and WRKY42 could form a transcriptional regulatory cascade in rice resistance to fungal pathogen [26]. WRKY13 could suppress WRKY42 expression by binding the W-box with the promoter region [26].

SolyWRKY Group III TFs not only responded to TYLCV infection by negative and positive regulation patterns, but also induced a series of defense protein expression (PR protein, chitinase) and hormone signaling (SA, abscisic acid, gibberellin acid) by binding *cis* elements [84]. SolyWRKY41 protein could interact with ICS protein by recognizing and binding W-box to regulate pathogen and stress response [70]. WRKY70 was demonstrated to be a common component in SA- and JA-mediated signal pathways [73]. Hormone signaling pathway would induce relative protein expressions, including proteinase inhibitors and thionin, to regulate secondary metabolism. Cross talk existed among signal transduction pathways, which could be important in plant immunity [78]. The extensive cross-regulation mechanism of WRKY Group III TFs is important in tomato–TYLCV defense.

## Conclusion

In this study, six WRKY Group III TFs in tomato were identified, and these TFs responded to TYLCV infection. Microsynteny revealed the independent duplication events, except whole-genome duplication, of WRKY Group III TFs. The phylogenetic tree of 28 species also showed that WRKY Group III TFs diversified after monocots and eudicots diverged. WRKY Group III TFs served as positive and negative regulators in tomato–TYLCV interaction. The expression levels of *SolyWRKY42* and *SolyWRKY80* were upregulated in ‘Zheza-301.’ In ‘Jinpeng-1,’ the expression patterns of the 6 WRKY Group III genes were positive. RT-qPCR and VIGS analyses indicated that *SolyWRKY41* and *SolyWRKY54* served as negative regulators in the resistance to TYLCV infection. WRKY Group III TFs could also interact with other proteins by binding to *cis* elements in the promoter region of other genes to regulate pathogen-related gene expression. Our study provided novel insights into the interaction of WRKY Group III TFs members with TYLCV infection in tomato.

## Additional files

Additional file 1:
**Table S1.** WRKY transcription factors name in rice, *Arabidopsis*, and tomato. Red color in tomato represented the SolyWRKY Group III TFs. **Table S2.** The orthologous WRKY genes in tomato, *Arabidopsis*, and rice. Red represented the orthologous genes of Group III genes responded to TYLCV infection in tomato. **Table S3.** The paralogous WRKY genes in tomato, *Arabidopsis*, and rice. Red represented the paralogous genes of Group III genes responded to TYLCV infection in tomato. (XLS 48 kb)
Additional file 2:
**Figure S1.** Structural analysis of WRKY transcription factors in tomato. A: Phylogenetic tree of SolyWRKY transcription factors. B: Exon/intrion structure analysis of WRKY transcription factors in tomato. (TIF 1982 kb)
Additional file 3:
**Figure S2.** Chromosome location of SolyWRKY TFs in tomato. Box represented the SolyWRKY Group III TFs in response to TYLCV infection. (TIF 5463 kb)

## Abbreviations

ABRE: *Cis*-acting element involved in abscisic acid response
CP: Coat protein
dsDNA: Double-stranded DNA
GFP: Green fluorescent protein
HSE: *Cis*-acting element involved in heat stress response
ICS: Isochorismate synthase
JA: Jasmonic acid
MAPK: Mitogen-activated protein kinase
MeJA: Methyl jasmonate
MS: Murashige and Skooog
OD: Optical density
ORF: Open reading frame
PAMPs: Pathogen associated molecular patterns
PCR: Polymerase chain reaction
PR: Pathogenesis-related
RT-qPCR: Quantitative real-time–polymerase chain reaction
SA: Salicylic acid
Soly: *Solanum lycopersicum*
ssDNA: Single-stranded DNA
TF: Transcription factor
TRV: Tobacco rattle virus
TYLCV: Tomato yellow leaf curly virus
VIGS: Virus-induced gene silencing.
