# Supplementary figures and images for: Members of WRKY Group III transcription factors are important in TYLCV defense signaling pathway in tomato (Solanum lycopersicum)
Source: BMC Genomics. 2016 Oct 7;17:788. doi: 10.1186/s12864-016-3123-2 (PMC5055730; doi:10.1186/s12864-016-3123-2)

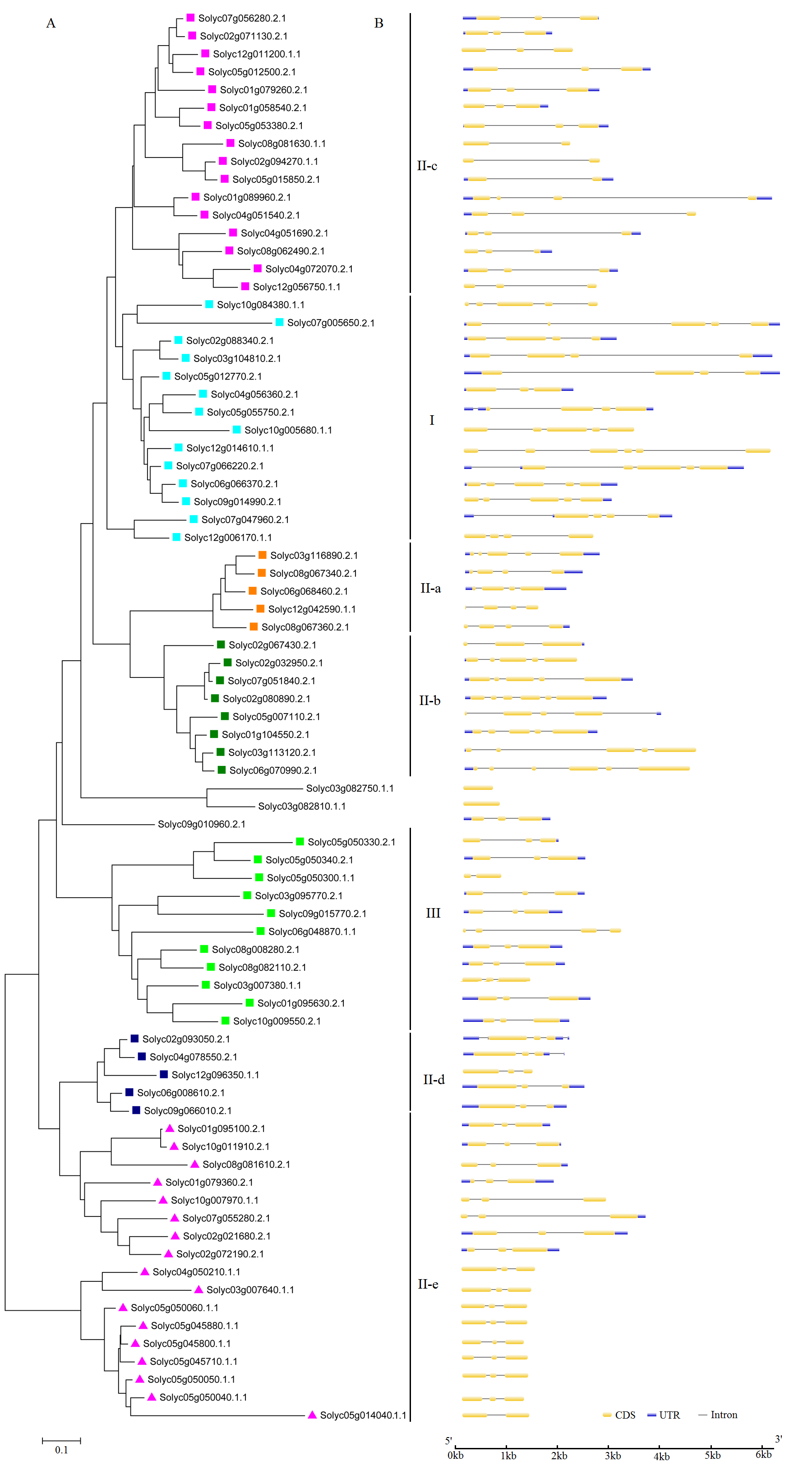

Supplement: Additional file 2: — Figure S1. Structural analysis of WRKY transcription factors in tomato. A: Phylogenetic tree of SolyWRKY transcription factors. B: Exon/intrion structure analysis of WRKY transcription factors in tomato. (TIF 1982 kb) [file 12864_2016_3123_MOESM2_ESM.tif]

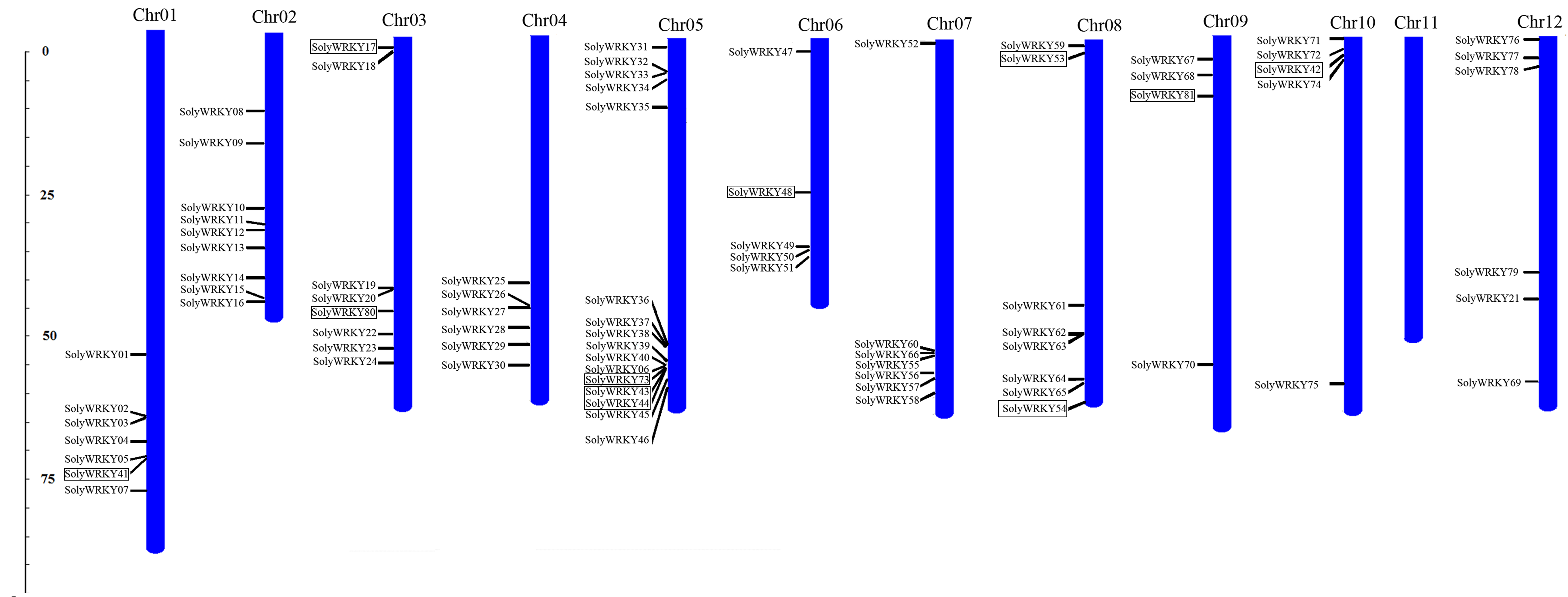

Supplement: Additional file 3: — Figure S2. Chromosome location of SolyWRKY TFs in tomato. Box represented the SolyWRKY Group III TFs in response to TYLCV infection. (TIF 5463 kb) [file 12864_2016_3123_MOESM3_ESM.tif]
